# A computable cellular stress network model for non-diseased pulmonary and cardiovascular tissue

**DOI:** 10.1186/1752-0509-5-168

**Published:** 2011-10-19

**Authors:** Walter K Schlage, Jurjen W Westra, Stephan Gebel, Natalie L Catlett, Carole Mathis, Brian P Frushour, Arnd Hengstermann, Aaron Van Hooser, Carine Poussin, Ben Wong, Michael Lietz, Jennifer Park, David Drubin, Emilija Veljkovic, Manuel C Peitsch, Julia Hoeng, Renee Deehan

**Affiliations:** 1 Philip Morris International R&D, Philip Morris Research Laboratories GmbH, Fuggerstr.3, 51149 Koeln, Germany; 2 Selventa, One Alewife Center, Cambridge, MA 02140, USA; 3 Philip Morris International R&D, Philip Morris Products S.A., Quai Jeanrenaud 5, 2000 Neuchâtel, Switzerland

## Abstract

**Background:**

Humans and other organisms are equipped with a set of responses that can prevent damage from exposure to a multitude of endogenous and environmental stressors. If these stress responses are overwhelmed, this can result in pathogenesis of diseases, which is reflected by an increased development of, e.g., pulmonary and cardiac diseases in humans exposed to chronic levels of environmental stress, including inhaled cigarette smoke (CS). Systems biology data sets (e.g., transcriptomics, phosphoproteomics, metabolomics) could enable comprehensive investigation of the biological impact of these stressors. However, detailed mechanistic networks are needed to determine which specific pathways are activated in response to different stressors and to drive the qualitative and eventually quantitative assessment of these data. A current limiting step in this process is the availability of detailed mechanistic networks that can be used as an analytical substrate.

**Results:**

We have built a detailed network model that captures the biology underlying the physiological cellular response to endogenous and exogenous stressors in non-diseased mammalian pulmonary and cardiovascular cells. The contents of the network model reflect several diverse areas of signaling, including oxidative stress, hypoxia, shear stress, endoplasmic reticulum stress, and xenobiotic stress, that are elicited in response to common pulmonary and cardiovascular stressors. We then tested the ability of the network model to identify the mechanisms that are activated in response to CS, a broad inducer of cellular stress. Using transcriptomic data from the lungs of mice exposed to CS, the network model identified a robust increase in the oxidative stress response, largely mediated by the anti-oxidant NRF2 pathways, consistent with previous reports on the impact of CS exposure in the mammalian lung.

**Conclusions:**

The results presented here describe the construction of a cellular stress network model and its application towards the analysis of environmental stress using transcriptomic data. The proof-of-principle analysis described here, coupled with the future development of additional network models covering distinct areas of biology, will help to further clarify the integrated biological responses elicited by complex environmental stressors such as CS, in pulmonary and cardiovascular cells.

## Background

The human body is constantly exposed to endogenous (e.g., mitochondrial reactive oxygen species (ROS) generation, unfolded protein response) and environmental stress. Stressors such as combustion products (diesel exhaust, carbon monoxide, nitrogen oxides, cigarette smoke), particulate matter, ozone, exert a daily challenge to our body's cellular defenses, in particular within the pulmonary and cardiovascular system [1,2]. Lung epithelial cells directly interface with the external environment and are often the first cells to be exposed to environmental stress [3,4]. While not facing the external environment directly, cells of the cardiovascular system are constantly exposed to the stressors that circulate in the bloodstream [5-7]. It is therefore not surprising that epidemiological studies have linked exposure to environmental stress to increased incidence of cardiovascular disease over the past decades [8-10]. Thus, further investigation into the mechanistic underpinnings of the response to different types of cellular stress is an important area of human health research [11-14].

One of the central challenges faced by contemporary investigators is how to comprehensively assess the biological impact of complex processes such as the cellular stress response at a molecular level, in order to understand their influence on disease susceptibility and progression. Computational approaches are increasingly being applied to analyze complex biological systems like the cellular stress response, including investigations into the role of key transcription factors such as NRF2 (mediating the antioxidative stress response), or identifying potential mechanisms for how stress can lead to diseases such as asthma [15,16]. Large scale, systems biology measurements (e.g., transcriptomics, proteomics, and metabolomics) can be applied to molecular regulatory network models in an effort to understand the underlying cellular response to biological insults. The field of pulmonary and cardiovascular biology has been quick to adopt systems biology approaches, using transcriptomic data to investigate the mechanistic basis behind the development of complex, multi-factorial diseases such as atherosclerosis and lung cancer [17-20], particularly with respect to the contribution of CS.

With a view to developing a Systems biology-based risk assessment approach for tobacco products, we are building a series of biological network models that reflect smoking-related molecular changes in the target tissues of the lung and the cardiovascular system. Detailed mechanistic networks are needed to drive the qualitative and eventually quantitative assessment of product-related data (conventional CS and harm reduced next generation products) to determine which pathways are activated in response to such exposures, and to measure the biological impact on in vitro and in vivo systems.

Physiological stress responses are diverse, depending on the type of stressor (chemical or physical), the tissue/cell types affected, and the duration and/or dose of the stressor. Therefore, in order to understand the biological pathways that are affected in response to a particular stressor in a specific physiological context, the availability of comprehensive network models that causally relate the relevant nodes (biological entities or processes) and edges (relationships between nodes) are needed to integrate systems biology data with the current knowledge of biological pathways. Ideally, the impact of environmental stress on all major cellular processes, e.g., proliferation, inflammatory processes, and apoptosis, can be evaluated by integrating multiple biological network models and systems biology data sets, using appropriate computational approaches. We have previously reported on the construction of a network model describing the pathways that are known to regulate cell proliferation in the lung as the first step towards the availability of a publicly available, integrated model of the major cellular processes operating in lung and cardiovascular tissues [21]. However, in order to holistically assess the effects of environmental and endogenous stressors on pulmonary and cardiovascular cells, as well as to link such effects to the onset of related diseases, the availability of detailed mechanistic network models for other major cellular processes is necessary.

Here we report the construction and testing of a more detailed network model reflecting the pathways that are described to operate in response to stress in non-diseased pulmonary and cardiovascular cells. Containing connectivity support from 428 unique literature sources, the network model conveys mechanistic detail about the pathways that are involved in response to several prominent pulmonary and cardiovascular cell stressors, including exogenous factors (i.e., air pollution, environmental toxicants) and endogenous factors (i.e., respiratory chain generated ROS, the unfolded/misfolded proteins). Model content boundaries were set to constrain the coverage of the network model to the stressors and stress responses that can occur in healthy, non-diseased cells of the pulmonary and cardiovascular systems. After establishing these content boundaries, we constructed a literature model of these processes. Next, we used computational analysis of four transcriptomic data sets to identify conserved sub-networks that are activated in response to different stressors, populating the network model with additional nodes and edges in the process.

Towards a verification of the network model, its descriptive content has to be assessed for correctness and relevance; therefore, the network model was evaluated for its ability to detect stress responses to a stressor that was not used to build the network model. Cigarette smoke (CS) contains thousands of chemicals that collectively induce complex molecular responses making CS an ideal test substance. The cellular response to stress induced by CS has been shown to be largely mediated by the oxidative-stress responsive transcription factor NFE2l2 (nuclear factor, erythroid derived 2, like 2; NRF2) making an NRF2 knockout mouse an ideal system to differentiate the response to stress using this network model [22,23]. Therefore, we tested the ability of the network model to detect cellular stress using transcriptomic data from mouse lung following acute *in vivo *CS exposure. In addition, we used the network model to investigate the response to acute CS exposure in mice that were constitutively deficient for NRF2. Our results suggest that the use of focused biological network models combined with large scale systems biology data sets can identify the salient biology underlying complex stressors like CS.

## Results

### Network Definition

#### Network model boundaries

The network model described here was constructed from content described from two sources, a literature model describing the relevant mechanisms involved in the stress response known from published literature, and a data set derived component, with content derived from the computational analysis of publicly available transcriptomic data from stress relevant experiments performed in pulmonary and cardiovascular cells. In order to ensure that the network model depicts biological mechanisms related to stress response in non-diseased pulmonary and cardiovascular tissues, we applied a set of rules for selecting network model content. Our overall goal was to generate a network model that reflects acute, non-pathological stress responses, and does not include the adjacent biological processes such as cell death/apoptosis, tissue damage, or inflammation which will be addressed in separate models.

Relationships derived from human tissue context were prioritized, however, if needed, connections derived from mouse and rat contexts were also used to complete the model (see Table 1 and Materials & Methods, "Knowledgebase" section). Canonical mechanisms representing pathways well-established in the literature were included in the network model even if literature support explicitly demonstrating the presence of the mechanism in lung- or cardiovascular-related tissues was not identified. For example, it was assumed that the same physiological machinery designed to combat metabolically generated ROS, e.g. the glutathione synthesis pathway, can operate in most mammalian cell types. However, if specific lung or cardiovascular contexts for canonical mechanisms were found in the literature, they were used. If needed to complete critical relationships within the network model, other tissue contexts were also considered, based on our assumption that they would reflect the response to stress in normal lung and cardiovascular tissues. For example, while liver contexts were generally excluded, they were used in the xenobiotic stress building block (see below for a description of building blocks) because many central mediators of xenobiotic stress response (e.g., AHR, PXR) have been extensively studied in hepatic systems. Additionally, renal contexts were generally excluded, with the notable exception of the osmotic stress building block, where renal cells are widely used as model systems to study osmotic regulation. Likewise, the use of causal relationships with tissue contexts from immortalized cell lines was limited to building critical mechanisms in the network model, when only available from this type of experimental system. In fact, causal relationships with tissue contexts derived from tumors or other diseased tissues were used at a frequency of only 1%. Since the Cellular Stress Network model is fully referenced, the tissue contexts for each causal edge are available for examination. Data derived from experiments with CS exposure were excluded during initial network building in order to maintain the ability to verify the network model at a later stage without bias from circularity.

**Table 1:** Summary of relevant statistics describing the content of the Cellular Stress Network model

Nodes	730
**mRNAs**	**84**

**Proteins**	**235**

**Phosphoproteins**	**43**

**Activities**	**180**

**Complexes**	**57**

**Protein families**	**18**

**Biological processes**	**48**

**Chemicals/Small molecules**	**65**

**Total Edges**	**1280**

**Causal Edges**	**778**

**Human-derived**	**545**

**Mouse-derived**	**175**

**Rat-derived**	**58**

**Unique PMIDs**	**428**

**Figure 1:**
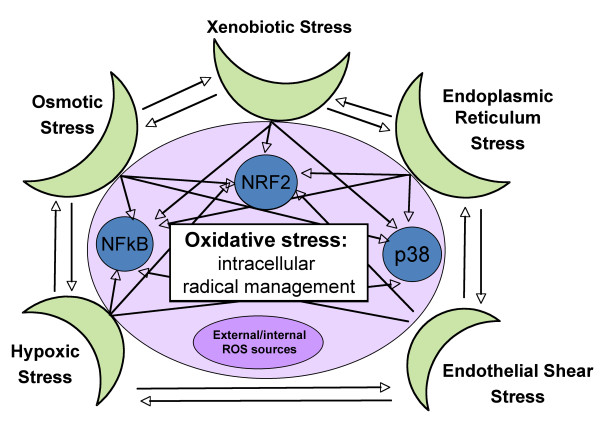
**Schematic overview of the modular "building block" framework used to construct the Cellular Stress Network**. A detailed network model of NRF2 signaling was included in the Oxidative Stress building block. A few examples of relevant transcription factors and kinase cascades included in the network model are shown.

Following an exhaustive search of the literature, components were selected for inclusion in the Cellular Stress Network model based on the biological mechanisms known to operate in response to stresses in lung and cardiovascular contexts, creating the mechanistic biological boundaries of the network model. The network model was constructed in a modular fashion using a "building block" framework in which the responses to several key types of stressors were modeled (see Figure 1). These building blocks contain overlapping nodes that, when joined, create an extensive network model of the pathways involved in the pulmonary and cardiovascular responses to physiological stress. The building blocks comprising the network model are:

##### Xenobiotic stress

Includes the role of AHR, Cytochrome p450 enzymes, and various environmental stressors.

##### Endoplasmic reticulum (ER) stress

Includes the unfolded protein response and the pathways downstream of the three key stress mediators: PERK (Eif2ak3), ATF6, and IRE1alpha (Ern1). The pro-apoptotic arm of the ER stress response was excluded from this network model in anticipation of being included in a separate network model on cell death related processes.

##### Endothelial shear stress

Includes the effects of laminar (atheroprotective) and turbulent (atherogenic) shear stress on monocyte adhesion, including NF-κB and nitric oxide pathways.

##### Hypoxic stress

Includes HIF1α activation and targets, control of transcription, protein synthesis, and crosstalk with oxidative stress, ER stress, and osmotic stress response pathways.

##### Osmotic stress

Includes NFAT5, aquaporin, and CFTR pathways downstream of the hyperosmotic response.

##### Oxidative stress

Includes intracellular free radical management, cellular responses to endogenous/exogenous oxidants and anti-oxidants and the glutathione metabolism. Key players of the involved intracellular pathways are the transcription factors AP-1, NF-κB and NRF2. A particular focus is on NRF2 as the central mediator of the cellular oxidative stress response including its upstream regulators and downstream gene expressions regulation via the antioxidant response element [24].

**Figure 2:**
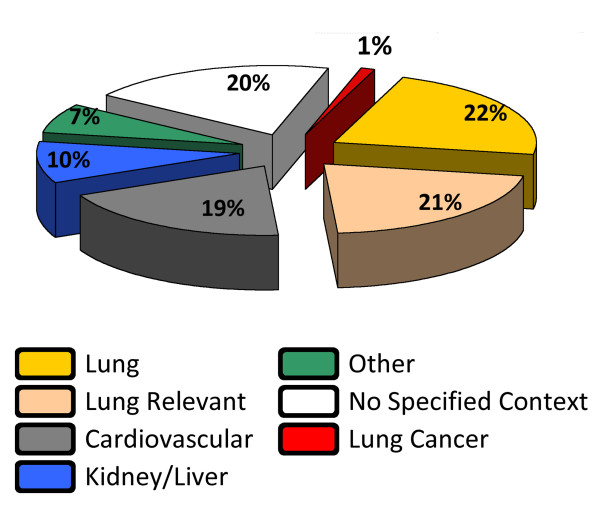
**Pie chart summarizing the tissue context origin of causal edges in the Cellular Stress Network (for details, see Additional File **1).

Ideally, all nodes and edges of the network model would be supported by published data from experiments conducted in non-diseased human, mouse, or rat pulmonary/cardiovascular tissue. However, in some cases, the results of the relevant detailed experiments have not been published. Thus, causal relationships with literature support coming from the tissues and cell types found in the normal lung (e.g., bronchial epithelial cells, alveolar type II cells, etc.) and in cardiovascular tissue (e.g., coronary artery endothelial cells) were prioritized. Approximately two thirds of the network model reflected lung and cardiovascular cell biology directly (Figure 2 and Additional File 1).

### Cellular stress network model literature component

The Cellular Stress Network model describes physiological stressors and the main processes operating in response to these stressors that occur in non-diseased lung and cardiovascular tissue. Specifically, this network model captures the responses to oxidative, endoplasmic reticulum, hypoxic, osmotic, xenobiotic, and shear stresses. Causal relationships (described in further detail in this section) describing these processes were added to the network model from the Selventa Knowledgebase [25], a unified collection of over 1.5 million elements of biological knowledge captured from the public literature and other sources. This network model was constructed using a computable framework, enabling its application to the evaluation of cellular stress based on systems biology data.

**Figure 3:**
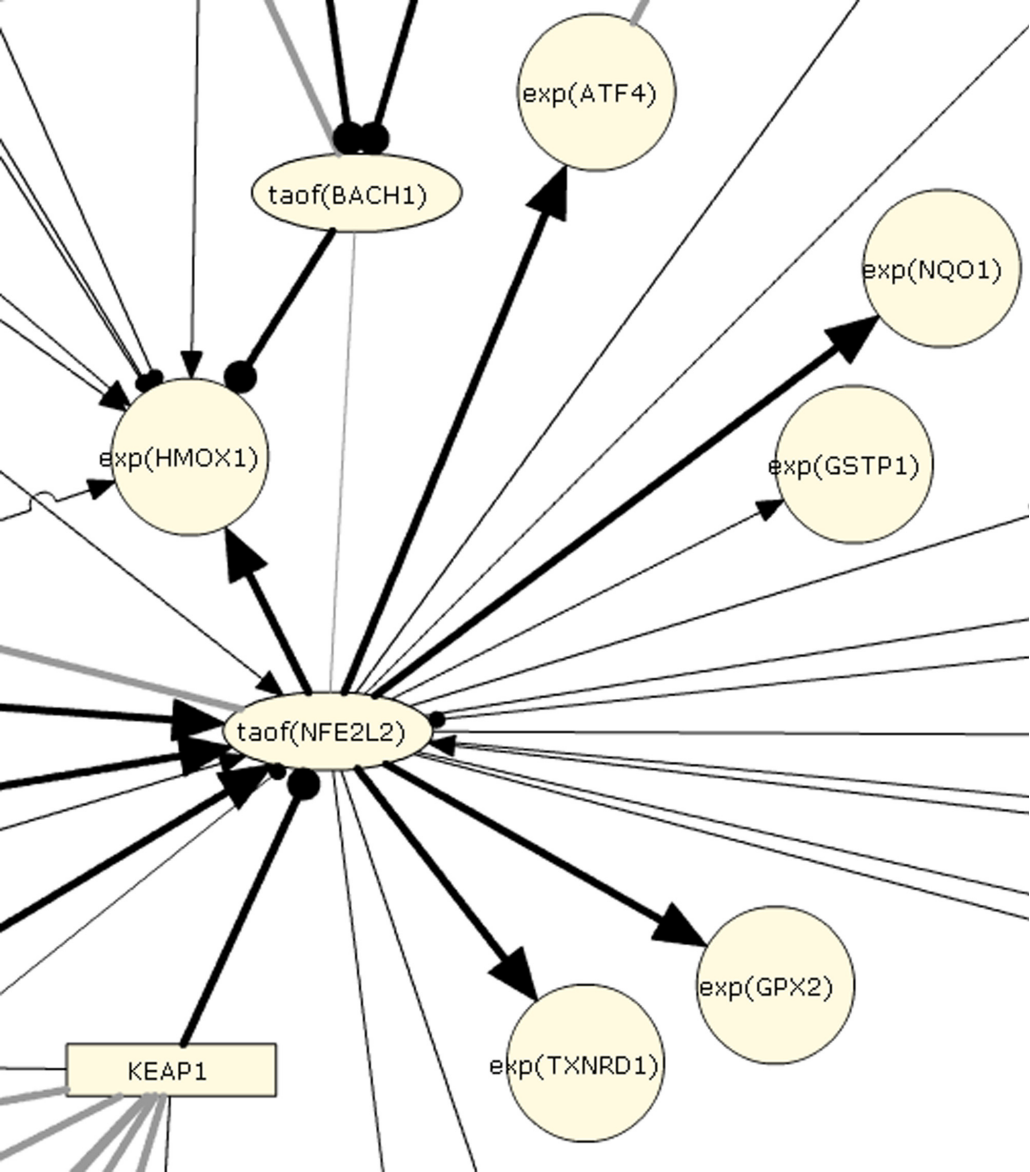
**Network model detail**. A portion of the network model surrounding NRF2 (NFE2L2) is shown, including transcriptional regulation by KEAP1 and downstream expression targets. Activating direct causal relationships are shown as dark arrows; inhibitory direct causal relationships are shown as edges ending in a knob.

**Figure 4:**
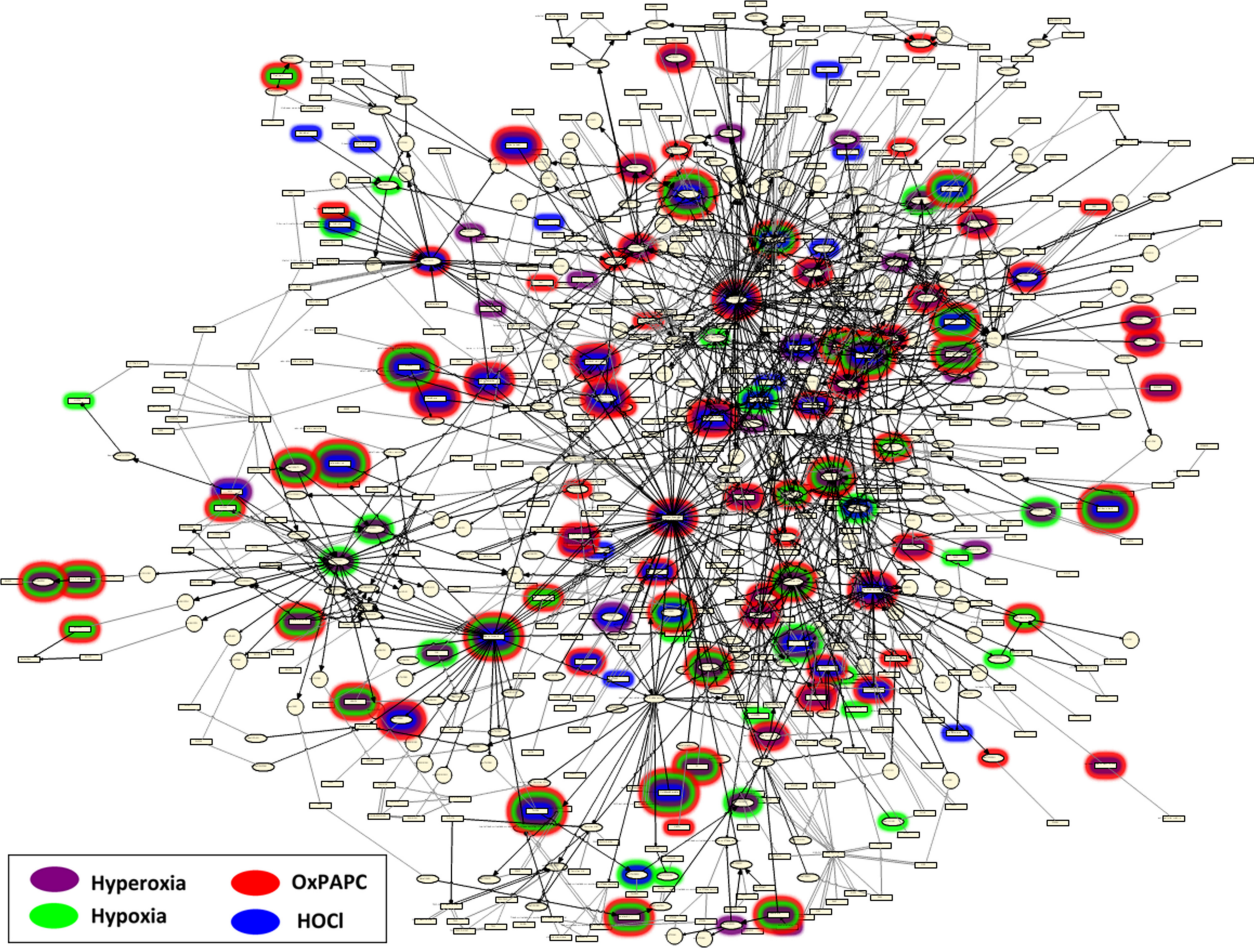
**The Cellular Stress Network**. Highlighted nodes are Reverse Causal Reasoning (RCR) hypotheses, predicted to have increased or decreased abundance or activity, in the indicated cell stress data sets.

The literature component of the Cellular Stress Network model contains 512 nodes and 876 edges. Network model nodes are biological entities such as mRNA expressions, protein abundances, or protein activities (Figure 3). Nodes may also be chemicals or small molecules whose transcriptional signatures may represent signaling similar to that which the chemical would induce. Finally, nodes can represent biological processes, such as "response to oxidative stress" or "laminar shear stress". This fine-grained representation allows for biological processes to be modeled with a high degree of mechanistic detail. Edges are relationships between nodes and may be either non-causal or causal. Non-causal edges simply connect different forms of a biological entity, such as its mRNA expression and its protein abundance, while causal edges are cause-effect relationships between biological entities based on primary literature data (Figure 4, for details, see Materials and Methods).

### Cellular Stress Network model data set component

#### Cell stress data sets

Application of Reverse Causal Reasoning (RCR, see below) to cellular stress transcriptomic data sets that capture the responses to a diversity of cellular stresses in lung and cardiovascular cell types was performed to confirm the activities of nodes already present in the literature portion of the network model, and also to supplement the literature-derived components of the network model with unique data set-derived nodes and edges. Data sets were selected with the goal of including a balance of mouse and human, *in vitro *and *in vivo *experiments, and a variety of cellular stresses. Data sets were selected to ensure representation from multiple building blocks, with oxidative stress as the focus. By using a variety of data sets which used different experimental stressors, we were able to confirm the literature-derived components in the network model and also add data set-derived nodes and edges from a variety of biological pathways, enhancing the breadth of the network model, in addition to its mechanistic detail. Furthermore, data sets with 48 hours or less treatment times were prioritized to best reflect the stress response mechanisms as they occur in non-diseased tissue. Other general data set selection criteria included: 1) how well physiologically-relevant stress in non-diseased lung or cardiovascular tissue was represented in the experiment, 2) the availability of phenotypic stress endpoint data, 3) the statistical rigor of the gene expression profiling experiments, and 4) the relevance of the experimental context to normal non-diseased lung or cardiovascular biology. The four data sets selected are summarized in Table 2. These data sets represent oxidative stress (Hyperoxia/GSE495 and HOCl/GSE15457), ER stress (OxPAPC/GSE20060), and hypoxic stress (Hypoxia/GSE11341). The Hyperoxia and Hypoxia experiments were performed in whole lung and a specific lung cell type, while the OxPAPC experiment was performed in a cardiovascular tissue context. Since the HOCl experiment was not performed in a lung or cardiovascular context, we assumed that the macrophage cell line used was generally reflective of the signaling that would occur in response to stress in lung macrophages as well.

**Table 2:** Data sets analyzed by RCR for assessment and augmentation of the Cellular Stress Network model

Data Set	Hyperoxia	HOCl	OxPAPC	Hypoxia
**Data Set ID**	**GSE495**	**GSE15457**	**GSE20060**	**GSE11341**

**PubMed ID**	**N/A**	**19376150**	**20170901**	**18469115**

**Perturbation**	**100% O**_**2**_	**1.4 mM hypochlorous acid**	**40 μg/ml oxidized phospholipid**	**1% O**_**2**_

**Tissue/Cells**	**Whole lung**	**RAW 267.4 cell line**	**Aortic endothelial cells**	**Lung microvascular endothelial cells**

***In vivo/in vitro***	***In vivo***	***In vitro***	***In vitro***	***In vitro***

**Species**	**Mouse**	**Mouse**	**Human**	**Human**

**Timepoint**	**48 h**	**6 h**	**4 h**	**48 h**

**Control**	**0 h**	**6 h untreated**	**4 h untreated**	**0 h**

**Platform**	**Affymetrix U74v2**	**Affymetrix 430_2**	**Affymetrix HGU133A**	**Affymetrix HGU133A**

**# State Changes**	**1122**	**1618**	**185**	**639**

**Measured outcome(s)**	**None**	**Cell viability, RT-PCR, and Western blot analysis of Nrf2 and Nrf2 target genes**	**qRT-PCR for HMOX1, GJA5**	**Hypoxia-induced marker genes; Scratch wound assay**

#### Reverse Causal Reasoning

Reverse Causal Reasoning (RCR) [25] was applied to identify statistically significant predictions of the activity states of biological mechanisms ("hypotheses") that are consistent with the measurements taken for a given systems biology data set. RCR on these four data sets identified upstream hypotheses which can explain the significant mRNA State Changes in each cell stress transcriptomic data set, enabling a deeper mechanistic understanding of the biological network perturbed by the experimental conditions, beyond the mere identification of significantly changing mRNAs [26,27]. These hypotheses represent mechanisms involved in the response to the various stressors used in the experiments. RCR prediction of activity for a given node using gene expression data sets requires a minimum of four observed RNA expression changes that are consistent with the predicted change in node activity. Thus, one reason that a network model node may not be predicted changed in the data sets is that the Knowledgebase contains too few causal connections from the node to downstream RNA expressions. To address this, we augmented the Selventa Knowledgebase with over 23,000 new statements from the public literature to enhance the prediction of nodes in the Cellular Stress Network model. Following this effort, 272 of the 730 nodes in the final Cellular Stress Network model were eligible for prediction (containing four or more downstream gene expression relationships and thus capable of prediction as a hypothesis) by RCR. As a notable caveat to these statistics, many of the nodes for which a prediction was not possible are "connector" nodes such as phosphorylations and complexes (145 nodes combined), which link protein activities to one another. For many of the predicted hypotheses, a corresponding literature-derived node was already present in the network model. Specifically, 43/272 (16%), 45/254 (18%), 23/163 (14%) and 30/246 (12%) RCR predicted HYPs were already nodes in the literature model for GSE495, GSE20060, GSE15457, and GSE11341, respectively. For example, RCR predicted the increased transcriptional activity of NF-κB in 3 out of the 4 data sets. Because the transcriptional activity of NF-κB was already in the literature model as a node, its prediction by RCR serves to verify its importance to the stress response, but did not add a new node to the network model.

#### Building block nodes are recapitulated by RCR results

RCR analysis on the four data transcriptomic data sets predicted the modulated activity or abundance for many nodes in the oxidative stress building block (Additional File 2). These include ROS and the transcriptional activity of NRF2, which are both predicted increased in each of the oxidative stress data sets (Hyperoxia and HOCl). Notably, there are also predictions for ER stress nodes in the ER stress data set (OxPAPC), such as increased "response to ER stress", Xbp1 transcriptional activity, and the activities of several ATF family members [28,29]. Finally, both the response to hypoxia and increased HIF1alpha activity hypotheses are predicted in the hypoxia data set. Hypotheses from the other building blocks of the Cellular Stress Network model are also predicted, including xenobiotic metabolism (AHR activity and the transcriptional signatures of the environmental contaminants tetrachlorodibenzodioxin, diesel exhaust, and soot), endothelial shear stress (laminar shear stress and monocyte adherence), and osmotic stress (NFAT5 activity, hyperosmotic response). Although these specific stresses did not have corresponding data sets, these predictions demonstrate the large degree of overlap between these stress response pathways.

#### Additional data set-derived nodes

For gap analysis and network augmentation, we further investigated those RCR-derived hypotheses from the four data sets that were not already represented in the literature network model. Thirty five hypotheses with clear impact on the response to cellular stress in the lung or cardiovascular tissues based on literature investigation of their biological roles were added to the network model. A table of these data set-derived hypotheses that were incorporated into the network model can be found in Additional File 3. The two-pronged approach of including both literature- and data set-derived nodes into the Cellular Stress Network model ensured that the network model covered a broad range of stress response pathways. This network model structure is critical to understanding complex stresses that can simultaneously activate multiple stress pathways.

For a complete list of nodes in each building block, see Additional File 4.

The final Cellular Stress Network model (a combination of the literature and data set derived components) contains 730 nodes and 1280 edges (778 of which are causal edges), and is supported by 428 unique PubMed-indexed references. This fully referenced Cellular Stress Network model is comprised of both literature-derived and data set-derived components (described in the subsequent sections) and provides the greater research community with the most comprehensive connectivity map of the molecular mechanisms involved in response to certain stresses in non-diseased lung and cardiovascular tissues currently in existence.

#### Cellular Stress Network model coverage

In total, 130 of the 272 RCR-capable network model nodes (48%) were predicted in at least one of the four data sets (Additional Files 5, 6, 7, 8). 83 (31%) were predicted based on the OxPAPC data set alone, while 72 (26%), 54 (20%) and 49 (18%) were predicted based on the Hyperoxia, Hypoxia, and HOCl data sets, respectively (Figure 4). These statistics are based on the full Cellular Stress Network model, including both literature-derived and data set-derived components. The presence of these hypotheses as nodes in the Cellular Stress Network model confirms that this network model is an accurate representation of the response to various physiological stresses in the lung and cardiovascular tissues. These hypotheses also confirm the ability of RCR to predict relevant biological mechanisms based on transcriptomic data from multiple, independent data sets. Therefore, this network model and the framework used to create it are well-suited for the evaluation of mechanisms involved in the response to cellular stress in the lung and cardiovascular tissues for a wide variety of relevant stressors.

### Cellular Stress Network model verification

To test the ability of the Cellular Stress Network model to provide qualitative mechanistic explanations for transcriptomic stress data, we investigated a recently published data series, GSE18344, which captures the transcriptional response to cigarette smoke (CS), as a prototypic inducer of pleiotropic cellular stress, in mouse lung [30]. This data series includes data from both wild type (WT) and NRF2 knockout (NRF2 KO) animals exposed to ambient air (sham exposure) or CS. The 1 day CS treatment data were chosen to test the Cellular Stress Network model; these data represent the stress response in non-diseased, naïve tissue that the network model was designed to evaluate.

**Figure 5:**
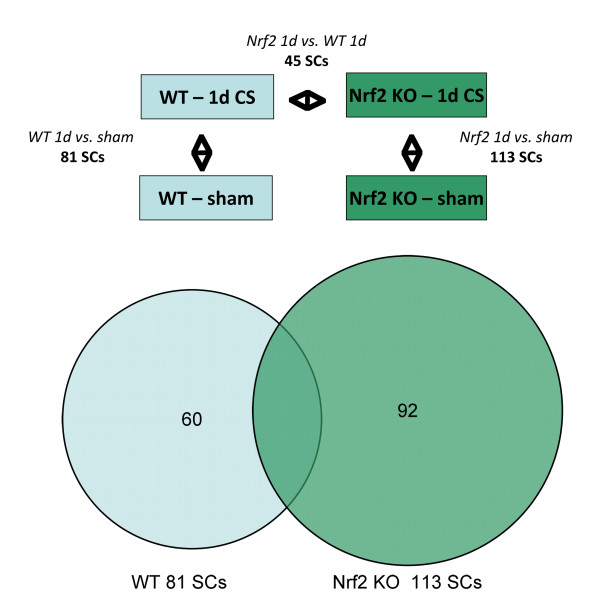
**Test data set and mRNA State Change overview**. (top) Test data set comparisons. Comparisons of GSE18344 data from 1 day cigarette smoke exposure experiments used to evaluate the Cellular Stress Network model. (bottom) mRNA State Change (SC) overlap between WT and NRF2 KO data sets. WT = wildtype mice; *NRF2 *KO = *NRF2 *knockout mice; SCs = mRNA State Changes.

Significant mRNA State Changes (SCs) were determined for three comparisons, (1) WT 1 day CS vs. sham exposure, (2) NRF2 KO 1 day CS vs. sham exposure, and (3) NRF2 KO 1 day CS vs. WT 1d CS exposure (Figure 5 and Table 3; see also Materials and Methods). In this analysis, an SC is a statistically significant difference in mRNA levels in different experimental conditions. The first two comparisons represent the response to 1 day CS exposure in WT and NRF2 KO mice, respectively. The third comparison represents the difference in response to CS in NRF2 KO compared to WT (Figure 5), and enables specific investigation of the contribution of NRF2 to the cellular response to CS. Because NRF2 is a key mediator of the cellular stress response in lung and other tissues [22,31,32], it is of great interest to compare the response to acute CS in WT and NRF2 KO mouse lungs. Notably, only 21 of 113 (19%) mRNA SCs induced by 1 day CS exposure in WT mice overlap with those observed in the NRF2 KO mice (Figure 5). These results are consistent with a central role for NRF2 in the lung cellular response to CS.

**Table 3:** Cellular Stress Network coverage statistics for the test data set comparisons based on GSE18344 data

Comparison	WT 1d vs sham	Nrf2 KO 1d vs sham	Nrf2 KO 1d vs WT 1d
**State Changes (SCs)**	**81**	**113**	**45**

**Significant Cellular Stress Network Hypotheses**	**39**	**47**	**23**

**SCs Explained by Network**	**67/81 (83%)**	**75/113 (66%)**	**40/45 (89%)**

**SCs Explained by Nfe2l2**	**29/81 (36%)**	**20/113 (18%)**	**31/45 (69%)**

**SCs Explained by Nfe2l2 or Keap1**	**37/81 (46%)**	**27/113 (24%)**	**36/45 (80%)**

SCs = mRNA State Changes; hypotheses - network model nodes predicted to have significantly increased or decreased activity by Reverse Causal Reasoning (RCR) on the SCs for each test data set comparison.

**Figure 6:**
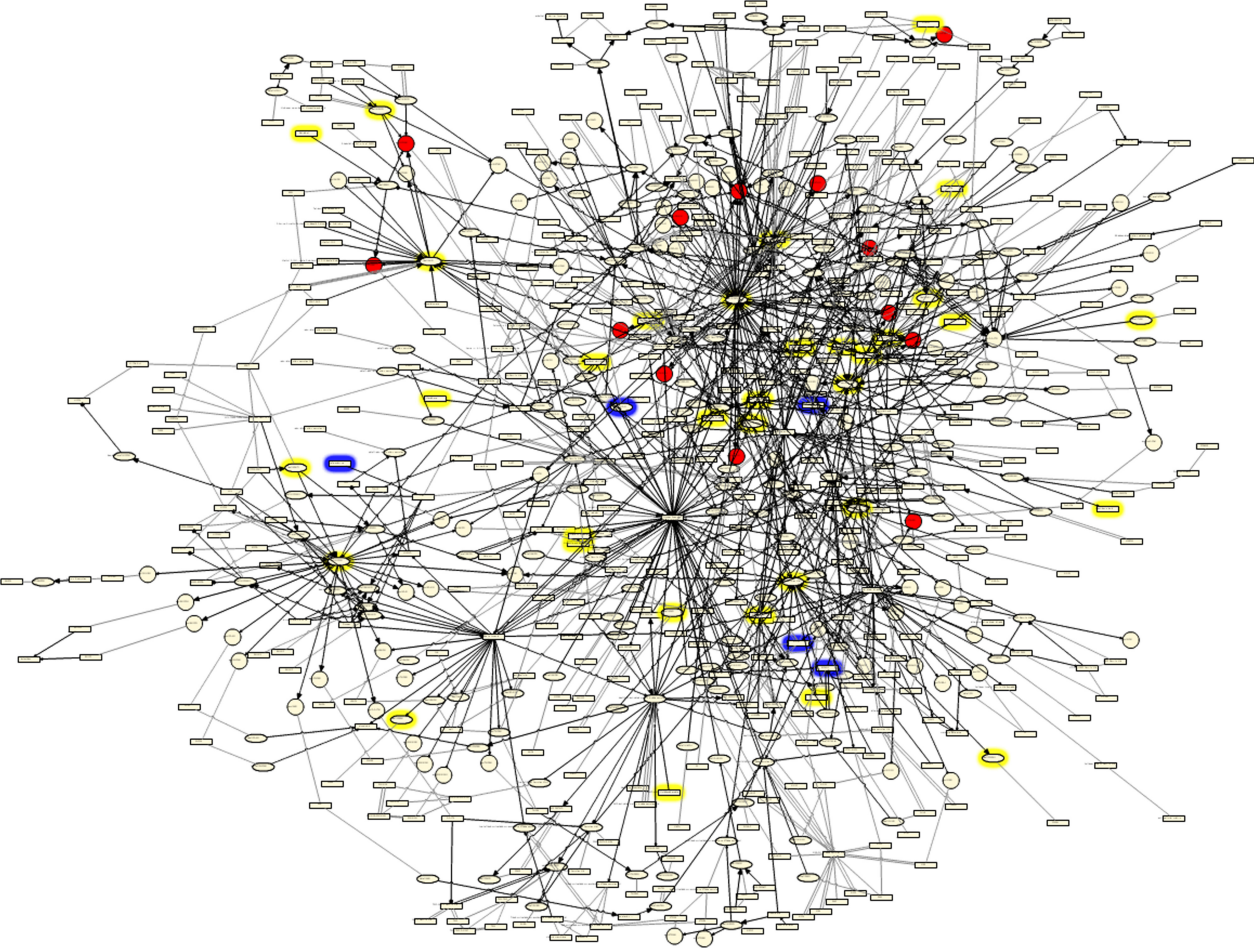
**Cellular Stress Network model colored for the WT 1 day cigarette smoke test data set**. Red - node corresponds to observed increased mRNA SCs; yellow halo - node is predicted by RCR to have increased activity; blue halo - node is predicted to have decreased activity.

RCR was performed on the significant mRNA SCs for each comparison to evaluate the ability of the Cellular Stress Network model nodes to explain the transcriptomic data (Additional File 9). Overlaying the significant hypothesis predictions and observed mRNA SCs from the WT 1 day vs. sham data set onto the network model (Figure 6) results in coverage of many network model areas, with a notable concentration of observed mRNA SCs around the transcriptional activity of NRF2 (taof(Nfe2l2)). Taken together, the significantly predicted hypotheses that are Cellular Stress Network model nodes explain 71/81 (88%) and 90/113 (80%) of the mRNA SCs induced by 1 day CS exposure in WT and NRF2 KO mice, respectively. The majority of SCs that were not explained by the Cellular Stress Network were those whose known upstream expression controllers fell outside of the network boundaries (e.g., IL18, NPAS1, TCF3). Future analyses of these data sets together with networks that describe other areas of CS-influenced biology such as inflammation, will serve to minimize these knowledge gaps.

Hypotheses significant in the WT or NRF2 KO 1-d CS data sets were placed into clusters based on their pattern of prediction in comparisons across all three CS data sets (Additional File 9). Cluster A is comprised of network model nodes predicted increased in WT 1-d vs. sham, and the opposite direction in the NRF2 KO 1-d vs. WT 1-d comparison, indicating signal dependence on NRF2. Cluster B is comprised of network model nodes predicted increased or decreased in the same direction for both the WT 1-d and NRF2 KO 1-d vs. sham exposure comparisons, but predicted in the opposite direction for the NRF2 KO 1-d vs. WT 1-d comparison, indicating that the signal is at least partially dependent on NRF2. Clusters A and B contain many components of the oxidative stress building block within the network model, including the oxidant hypotheses "Hypochlorous acid" and menaquinone ("Menadione"), as well as NRF2 ("Nfe2L2") itself and its negative regulator, "Keap1". Cluster C is comprised of nodes predicted increased in both WT 1-d vs. sham and NRF2 KO 1-d vs. sham, with no predicted differences in the NRF2 KO 1-d vs. WT comparison. These nodes come from a mix of network model building blocks and include the ER stress-inducer "Tunicamycin" as well as "ATF6", a transcription factor activated by the unfolded protein response [33]. Cluster D is comprised of nodes predicted up- or down-regulated by CS exposure in WT 1-d and not the NRF2 KO 1-d, but with no significant difference between WT 1-d and the NRF2 KO 1-d when directly compared. Cluster E is comprised of nodes predicted changed in the NRF2 KO 1-d vs. sham comparison only. While clusters A and B represent elements of the stress response influenced by NRF2, cluster C represents likely NRF2-independent components of the stress response. Most of the network model nodes from the oxidative stress building block are present in the NRF2-influenced clusters A and B, consistent with the key role for NRF2 in the oxidative stress response.

Notably, 29/81 (35%) of SCs induced by 1 day CS exposure in WT mice can be explained by activation of NRF2. Expanding this calculation to include KEAP1, a negative regulator of NRF2 and key mediator of its activation by oxidative stress [34], explains 37/81 (46%) of the WT 1 day vs. sham SCs. While the NRF2 KO mice lack NRF2, 20/113 (18%) SCs induced by CS exposure can be explained by NRF2, and 27/113 (24%) explained by NRF2 and KEAP1 network model nodes together. Some of the genes that can potentially be controlled by NRF2 can also be controlled by other, NRF2-independent mechanisms [35-37]. When the 1 day CS exposed NRF2 KO mice are compared to the WT mice, decreased transcriptional activity of NRF2 is predicted, consistent with the absence of NRF2 in these mice.

## Discussion

### The Cellular Stress Network model is a unique resource

The Cellular Stress Network model was designed to be used as a comprehensive research resource for the scientific community and as a functional backbone for computational analysis. As a publicly available research resource, the network model can be used by investigators to explore the connectivity of the genes/proteins/processes involved in different stress responses relevant to their research programs. Until now, no such single resource existed for the pulmonary and cardiovascular research communities. In addition, the network model is compatible with computational reasoning to analyze systems biology data.

One unique aspect of the Cellular Stress Network model is its specificity with respect to tissue context. We focused network model connectivity on mechanisms that operate in a defined set of cell types relevant to cardiovascular and pulmonary biology. Other common approaches for building connectivity networks that integrate prior knowledge, e.g., using Kyoto Encyclopedia of Genes and Genomes (KEGG) maps or protein-protein interaction databases, generally compile connections that have been reported in many different tissue types, sometimes in the context of disease as an added advantage over other common pathway analyses, the edges in the network model presented are embedded with accessible literature evidence supporting each relationship, making for a highly transparent network model. Last, because the edges in the network model described here are supported by causal relationships directly observed in published experiments, the network model contains a unique level of biological transparency.

The Cellular Stress Network model is part of a broader systems biology initiative. Previously, we reported on the construction and utility of a network model describing pathways known to be involved in regulating cell proliferation in the non-diseased lung (Cell Proliferation Network model) [21]. Additional biological process network models, constructed using a similar modular design, can then be combined with the existing Cell Proliferation and Cellular Stress Network models. Forming an integrated network that covers an unparalleled level of complex pulmonary and cardiovascular-related biology, this collection of network models will be an invaluable resource to the greater research community, aiding in the effort to understand the underpinning mechanisms. Eventually, this integrated network will serve as a scaffold for the parallel analysis of multiple systems biology data types (e.g., phosphoproteomics) in combination with transcriptomic data to assess complex biology.

Other lung-focused stress networks have been generated using systems biology data (specifically gene expression profiling), however they differ in their construction methods, content, applications, and explanatory power. For example, Freishtat et al. report a 26-member lung stress network comprised of genes regulated by asthma-relevant challenges or tobacco smoke in multiple gene expression data sets [16]. A second example network used information-theoretic network inference algorithms to identify NRF2 targets and regulatory relationships using a large number of mouse lung microarray data sets [15]. Similar to the Cellular Stress Network model reported here, these networks are relevant to the stress response in lung tissue and make use of microarray data for their construction; however, these networks differ in that they have highly focused application and less explanatory power for experimentally observed gene expression changes. The relatively large size and comprehensive biological coverage of the Cellular Stress Network model imparts it with a unique ability to explain systems biology data and provide mechanistic detail.

### The Cellular Stress Network model captures diverse stress responses in pulmonary and cardiovascular cells

The daily environmental assaults posed to normal pulmonary and cardiovascular cells can exert multiple, complex, and often interconnected stress responses. In order to unravel the mechanisms behind these integrated responses using systems biology data sets (e.g., gene expression profiling), the Cellular Stress Network model was designed to represent the response to stress in normal, non-diseased lung and cardiovascular cells. To focus the network model on this tissue-specific stress response, we used four data sets representing some of the stresses that lung and cardiovascular cells are exposed to. These data sets not only provided a means to assess the content of the literature-derived portion of the network model, but perhaps more importantly, revealed the shared and unique mechanisms that operate in pulmonary and cardiovascular cells following exposure to stress. The hypoxia data set aided in ensuring the hypoxia response signaling was comprehensively captured in the network model. Similarly, the hyperoxia and HOCl (inducers of oxidative stress [38-40]) data sets aided in construction and evaluation of the oxidative stress response mechanisms in the network model. OxPAPC, a pro-inflammatory oxidized phospholipid that induces both oxidative and ER stress [41], provided a fourth stress data set to aid network model construction. These data sets come from a variety of lung and cardiovascular-relevant tissues from both human and mouse, as well as both *in vivo *and *in vitro *stressors. The network model construction strategy of using data sets together with literature-derived tissue-specific and canonical pathway mechanisms ensured that the network model provides comprehensive coverage of a range of physiological and environmental stressors affecting the lung and cardiovascular system, a critical aspect of a network model designed to evaluate integrated stress responses.

Several network model nodes were predicted by RCR to increase or decrease in activity across multiple data sets. The responses to the different types of stress represented by the Cellular Stress Network model are integrated - while the stressors and some response pathway elements are unique, many common signaling pathways are shared. The structure of the Cellular Stress Network model as a collection of nodes linked by edges representing qualitative relationships between the nodes captures causal connectivity between response pathways for different stresses. For example, while NRF2 is a key regulator of the oxidative stress response, it can be activated by other stressors. ER stress activates NRF2 through phosphorylation by Eif2ak3 (PERK) [42], shear stress activates it via Klf2 or 15-deoxy-Δ(12,14)-prostaglandin J2 [43,44], and intermittent hypoxia and xenobiotic metabolism stress activate NRF2 through activation of ROS production [45,46]. Notably, NRF2 activation is predicted by RCR in three of the four data sets used to guide network model construction: OxPAPC, hyperoxia, and HOCl. Similarly, the transcriptional activity of the NF-κB complex is activated by multiple stresses, including oxidative, shear, ER, and hypoxic stress [47-51], and is predicted to have increased activity in three of the four data sets: hypoxia, OxPAPC, and hyperoxia. These points of stress signaling integration are captured in detail by the network model, facilitating the application of the network model to the analysis of complex stressors which may activate multiple signaling pathways.

### The Cellular Stress Network model can be used with systems biology data to identify mechanistic explanations for complex cellular responses

One of the benefits of using systems biology analyses, like transcriptomic profiling, is the wealth of data that is provided following experimental application of a stressor. For contemporary scientists, a modern challenge is how to transform this biological data into meaningful mechanistic explanations for the observed biology following experimental stress induction. This is especially challenging for the cellular stress response, which can manifest in complex, overlapping signaling responses. We tested the Cellular Stress Network model by applying it to the analysis of gene expression profiling data for the response to acute CS exposure in WT mouse lung (GSE18344;[30]). The Cellular Stress Network model explained 88% of the mRNA SCs induced by CS in WT. Notably, a significant portion of these SCs (46%) can be explained by the oxidative and electrophilic stress-activated transcription factor NRF2 or its negative regulator KEAP1. Our results, consistent with the reported role of NRF2 in the *in vivo *lung response to CS [52], provide additional confidence in the ability of the Cellular Stress Network model to identify stress pathways using transcriptomic data.

In addition to NRF2, other elements of the Cellular Stress Network model predicted to be activated in WT mice by acute CS exposure include the response to ER stress and the ER stress response-induced transcription factors ATF4 and ATF6. Moreover, the oxidative stress building block network model components "gtpof(Kras)" and "taof(AP-1 complex)" are predicted activated in response to CS. These elements are predicted in both WT and NRF2 KO mice, and are not differential in the direct comparison of the NRF2 KO to WT mice, suggesting that this response is NRF2-independent. In addition, these predictions are consistent with previous reports of CS-induced signaling mechanisms.

CS has been reported to induce ER stress in both diseased and non-diseased lung cells [53,54] as well as in other cell types [55]. Moreover, CS has been reported to induce the proteolytic cleavage and activation of ATF6 as well as the increased nuclear expression of ATF4 in cultured human lung cells in response to acute CS exposure [53,54]. While ATF4 physically interacts with NRF2 [56], the prediction of increased ATF4 in both WT and NRF2 KO mice in response to CS suggests that NRF2 is not required for ATF4 transcriptional activity.

Similar to the ER stress response, KRAS and AP-1 activation represent portions of the stress response that are activated by acute CS exposure that are not dependent on NRF2. These oxidative stress response mechanisms are predicted activated in both WT and NRF2 KO mice. AP-1 has been implicated in CS-induced gene expression in lung [57,58] and in Swiss 3T3 cells [59]. ROS have been demonstrated to activate RAS family members in a variety of tissues including the lung and cardiovascular-relevant cell types, fibroblasts and smooth muscle cells [60-62].

We report here both the construction of a literature-based network describing cellular stress signaling in the lung, and the assessment of cellular stress signaling in this network for several RNA expression data sets. Our approach for assessing pathway activation utilizes RCR, where the differential mRNA expression of genes is used to infer the activity of nodes/pathways in the network based on causal relationships. Several other methodologies for detecting pathway activation using transcriptomic profiling data as a substrate have been published previously. One common approach is to generate interaction (protein-protein, protein-gene) networks from publicly available resources (databases, published experiments, etc.) [63-66]. Using these interaction networks, differentially expressed genes from an experimental test case are then used to identify statistically enriched pathways or subnetworks. Here, protein subnetworks are identified on the basis of the structured expression patterns of their genes (i.e. subnetworks are identified if the genes encoding the proteins in a subnetwork are all observed to increase or decrease) in a stereotyped fashion. In contrast, we use the differentially expressed genes in the context of prior knowledge-derived causal relationships between the genes and their upstream controllers to infer pathway activity.

## Conclusions

The cellular response to stress is a key process mediating adaptation and survival, particularly in tissues like lung with significant direct environmental exposure. Systems biology data such as gene expression profiling hold great promise for the comprehensive assessment of complex molecular signaling processes like the cellular response to stress. The non-diseased lung and cardiovascular tissue-focused Cellular Stress Network model described here is a fully referenced mechanistic representation of multiple physiological stress response pathways, including oxidative stress, ER stress, and the response to hypoxia. The adaptable and computable structure of this network model provides a useful framework for assessing and investigating biological impact from systems biology data. When tested using lung-derived transcriptomic data from CS-exposed mice, it explained a large proportion (88%) of the observed significant mRNA expression changes, and mechanistically confirmed the role of NRF2, a known mediator of the oxidative stress response, as a central contributor to the CS-induced stress response.

## Methods

### Knowledgebase

The nodes and edges comprising the Cellular Stress Network model were added to the model from the Selventa Knowledgebase, a repository containing over 1.5 million nodes (biological concepts and entities) and over 7.5 million edges (connections between nodes). The Selventa Knowledgebase is comprised of causal and non-causal assertions between biological entities or processes derived from peer-reviewed scientific literature as well as other public and proprietary databases. Causal assertions are derived from published literature reporting on experiments performed in human, mouse, and rat species contexts, both *in vitro *and *in vivo*. Causal assertions also capture additional details about the relationship and tissue context in which the relationship was experimentally observed to occur. Notably, correlative relationships, particularly from clinical studies, are also captured in the Knowledgebase. Each causal assertion is associated with its source information as well as key information including the species (human, mouse, or rat) and the tissue or cell line from which the experimental observation was derived. An example causal assertion is the increased transcriptional activity of Ahr (aryl-hydrocarbon receptor) causes an increase in the mRNA expression of Cyp1a1 (cytochrome P450, family 1, subfamily a, polypeptide 1). Causal assertions are encoded using Biological Expression Language (BEL), an intuitive language developed at Selventa that provides a framework for qualitative modeling of biological processes. BEL enables the development of computable pathway models comprised of cause and effect relationships, as well as construction of knowledgebases of biological relationships suitable for automated reasoning methods such as Reverse Causal Reasoning (RCR, see Materials and Methods below). The assembled collection of these causal assertions is referred to as either the human or mouse Knowledge Assembly Model (KAM). The Knowledgebase contains causal relationships derived from healthy tissues and disease areas such as inflammation, metabolic diseases, cardiovascular injury, liver injury, and cancer.

### Analysis of transcriptomic data sets

Four previously published cell stress data sets, GSE495 (hyperoxia), GSE15457 (HOCl), GSE20060 (OxPAPC), and GSE11341 (hypoxia), were used for the construction of the Cellular Stress Network model (Table 2). GSE18344 (CS) was used for Cellular Stress Network model testing. All five data sets were downloaded from Gene Expression Omnibus (GEO) http://www.ncbi.nlm.nih.gov/gds. Raw RNA expression data for each data set were analyzed using the "affy" and "limma" packages of the Bioconductor suite of microarray analysis tools available for the R statistical environment [67-70]. Robust Microarray Analysis (RMA) background correction and quantile normalization were used to generate microarray expression values. An overall linear model was fit to the data for all sample groups, and specific contrasts of interest were evaluated to generate raw *p*-values for each probe set on the expression array [71]. The Benjamini-Hochberg False Discovery Rate (FDR) method was then used to correct for multiple testing effects.

Probe sets were considered to have statistically significant changed expression levels in a specific comparison if they had an adjusted *p*-value of lower than 0.05 and an absolute fold change greater than 1.3. An additional expression abundance filter was applied to three of the data sets; probe set differences were considered significant only if the average expression intensity was above 250. NetAffx version na31 feature annotation files, available from Affymetrix http://www.Affymetrix.com, were used for mapping of probe sets to genes. In our analysis, genes represented by multiple probe sets were considered to have changed if at least one probe set was observed to change. Gene expression changes that met these criteria are called "State Changes" and have the directional qualities of "increased" or "decreased", i.e., they were upregulated or downregulated, respectively in response to the experimental condition. The number of State Changes for each data set is listed in Table 2.

### Reverse Causal Reasoning (RCR): Automated hypothesis generation

RCR of the four cell stress transcriptomic data sets was used to aid in the selection of nodes for the Cellular Stress Network model. RCR interrogates a Knowledge Assembly Model to identify upstream controllers of the RNA State Changes observed in the data set (see [25] for specific detail on RCR). For the hypoxia and OxPAPC data sets, the human KAM was used, while the mouse KAM was used for the HOCl, hyperoxia, and CS data sets. These potential upstream controllers identified by RCR are called "hypotheses", as they are statistically significant potential explanations for the observed RNA State Changes.

Each hypothesis is scored according to two probabilistic scoring metrics, richness and concordance. Richness is the probability that the number of observed RNA State Changes connected to a given hypothesis could have occurred by chance alone, calculated using the hypergeometric distribution. Concordance is the probability that the number of observed RNA State Changes that match the direction of the hypothesis (e.g., increased or decreased activity or abundance of a node) could have occurred by chance alone, calculated using a binomial distribution. Hypotheses meeting both richness and concordance p-value cutoffs of 0.1 were considered to be statistically (although not necessarily biologically) significant. For the purposes of network model construction, each scored hypothesis meeting the minimum statistical cutoffs for richness and concordance was evaluated and selected for integration based on its biological plausibility and relevance to the experimental stress used to generate the data.

Additional File 10 shows the color key and abbreviations for the tables in this section, while Additional File 3 shows all of the hypotheses predicted by RCR on the four data sets that were present in the Cellular Stress Network model. These hypotheses may also be visualized in Figure 4, which is a schematic diagram of the Cellular Stress Network model with the hypotheses predicted in each of the four cellular stress data sets identified by colored halos around the hypothesis node. The Cellular Stress Network accompanies this manuscript in.xls (Additional File 11) and.owl (Additional File 12) formats, and can be viewed using freely available network visualization software such as Cytoscape http://www.cytoscape.org/.

## Abbreviations

AHR: aryl hydrocarbon receptor; BEL: Biological Expression Language; CS: cigarette smoke; ER: endoplasmic reticulum; FDR: false discovery rate; GEO: Gene Expression Omnibus; HIF1A: hypoxia inducible factor 1, alpha subunit; Hmox1: heme oxygenase (decycling) 1; HOCl: hypochlorous acid; KEGG: Kyoto Encyclopedia of Genes and Genomes; KO: knockout; mRNA: messenger ribonucleic acid; Nfe2l2: nuclear factor, erythroid derived 2, like 2 (NRF2); Nqo1: NAD(P)H dehydrogenase [quinone] 1; OxPAPC: oxidized 1-palmitoyl-2-arachidonoyl-sn-glycero-3-phosphocholine; PMID: PubMed identifier; Prdx1: Peroxiredoxin-1; PXR: pregnane X receptor; RCR: Reverse Causal Reasoning; RMA: Robust Microarray Analysis; RNA: ribonucleic acid; ROS: reactive oxygen species; SC: State Change; WT: wild-type.

## Competing interests

Selventa and PMI authors performed this work under a joint research collaboration funded by PMI.

## Authors' contributions

WKS contributed to the network design, biological content, interpretation of results, manuscript revision, and project co-ordination. JWW contributed to the network design, biological content, interpretation of results, and manuscript revision. SG, NLC, CM, BPF, AH, CP, ML, EV contributed to the network design, biological content, interpretation of results, and manuscript preparation. DD, AAVH, BW, JP contributed to the biological content and interpretation of results. MCP, JH contributed to system concept and supervised the project. RD contributed to system concept, network design, interpretation of results, manuscript preparation and supervised the project. All authors read and approved the final manuscript.

## Supplementary Material

Additional file 1 **Tissue context origins for causal edges in the Cellular Stress Network**. Corresponding tissue context categories are referenced in Figure 2. Click here for file

Additional file 2 **RCR-predicted hypotheses in the Cell Stress Network model**. Indicates nodes that are RCR-predicted hypotheses from the four cell stress data sets analyzed (Hypoxia, OxPAPC, Hyperoxia, and HOCl). The building block(s) in which these nodes are contained is also shown in the rightmost column. See Additional File 10 for color and abbreviation key. Click here for file

Additional file 3 **Data set-derived nodes added to the Cellular Stress Network based on their predictions as hypotheses**. See Additional File 10 for color and abbreviation key. Click here for file

Additional file 4 **Tables showing the nodes contained in each building block that comprise the Cellular Stress Network**. Click here for file

Additional file 5 **Cellular Stress Network model colored for the HOCl data set**. Red - node corresponds to observed increased mRNA; yellow halo - node is predicted by RCR to have increased activity; blue halo - node is predicted to have decreased activity. Click here for file

Additional file 6 **Cellular Stress Network model colored for the hyperoxia data set**. Red - node corresponds to observed increased mRNA; green - node corresponds to observed decreased mRNA; yellow halo - node is predicted by RCR to have increased activity; blue halo - node is predicted to have decreased activity. Click here for file

Additional file 7 **Cellular Stress Network model colored for the hypoxia data set**. Red - node corresponds to observed increased mRNA; green - node corresponds to observed decreased mRNA; yellow halo - node is predicted by RCR to have increased activity; blue halo - node is predicted to have decreased activity. Click here for file

Additional file 8 **Cellular Stress Network model colored for the OxPAPC data set**. Red - node corresponds to observed increased mRNA; green - node corresponds to observed decreased mRNA; yellow halo - node is predicted by RCR to have increased activity; blue halo - node is predicted to have decreased activity. Click here for file

Additional file 9 **RCR-predicted Cellular Stress Network model hypotheses for the test data set comparisons**. Hypotheses are grouped by pattern of prediction across the three test data set comparisons. See Additional File 10 for color and abbreviation key. Click here for file

Additional file 10 **Color and abbreviation key for hypothesis nodes**. Click here for file

Additional file 11 **The Cellular Stress Network,.xls format**. Click here for file

Additional file 12 **The Cellular Stress Network,.owl format**. This file can be viewed using freely available network visualization software such as Cytoscape http://www.cytoscape.org/. Click here for file
