# Supplementary figures and images for: A computable cellular stress network model for non-diseased pulmonary and cardiovascular tissue
Source: BMC Syst Biol. 2011 Oct 19;5:168. doi: 10.1186/1752-0509-5-168 (PMC3224482; doi:10.1186/1752-0509-5-168)

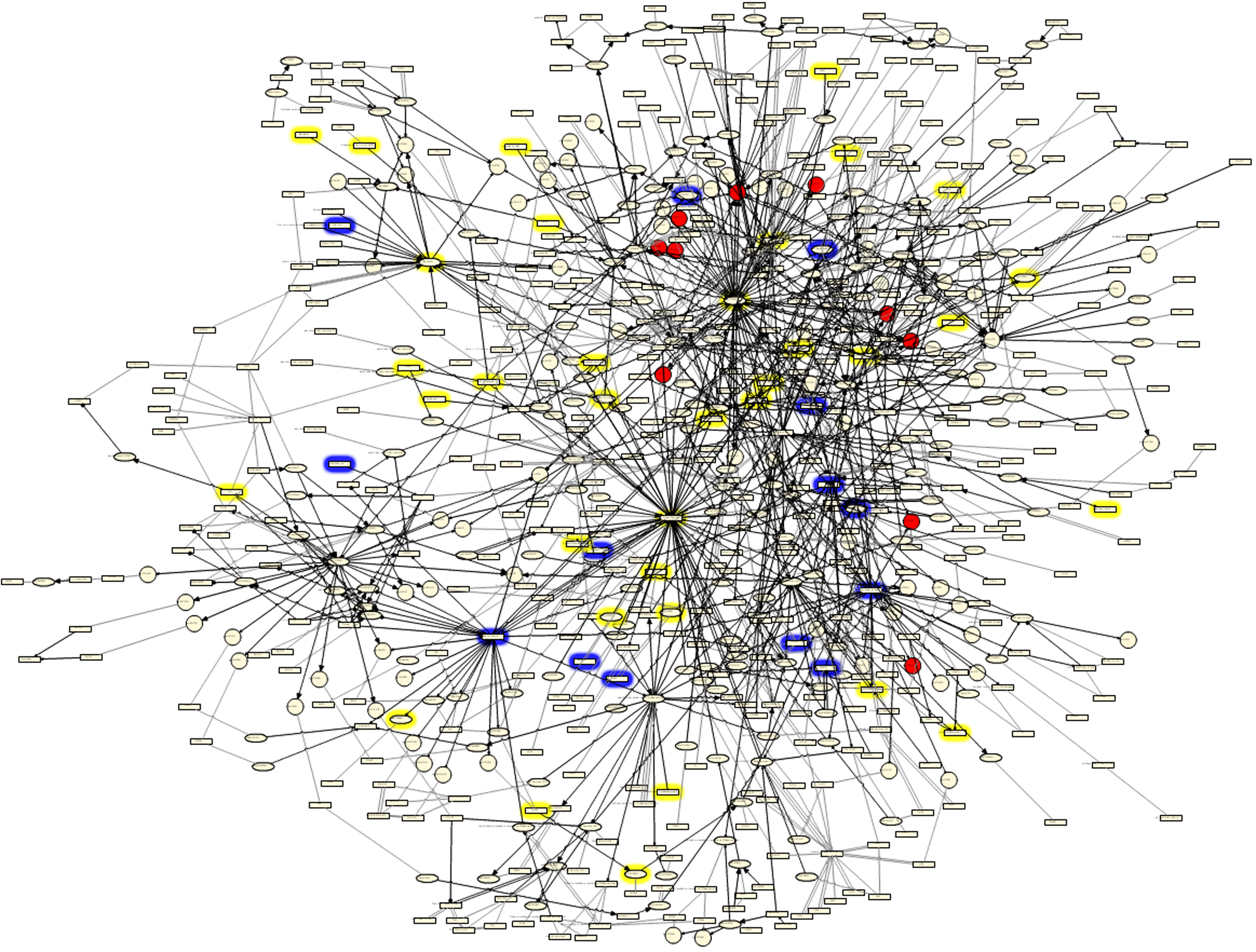

Supplement: Additional file 5 — Cellular Stress Network model colored for the HOCl data set. Red - node corresponds to observed increased mRNA; yellow halo - node is predicted by RCR to have increased activity; blue halo - node is predicted to have decreased activity. [file 1752-0509-5-168-S5.PNG]

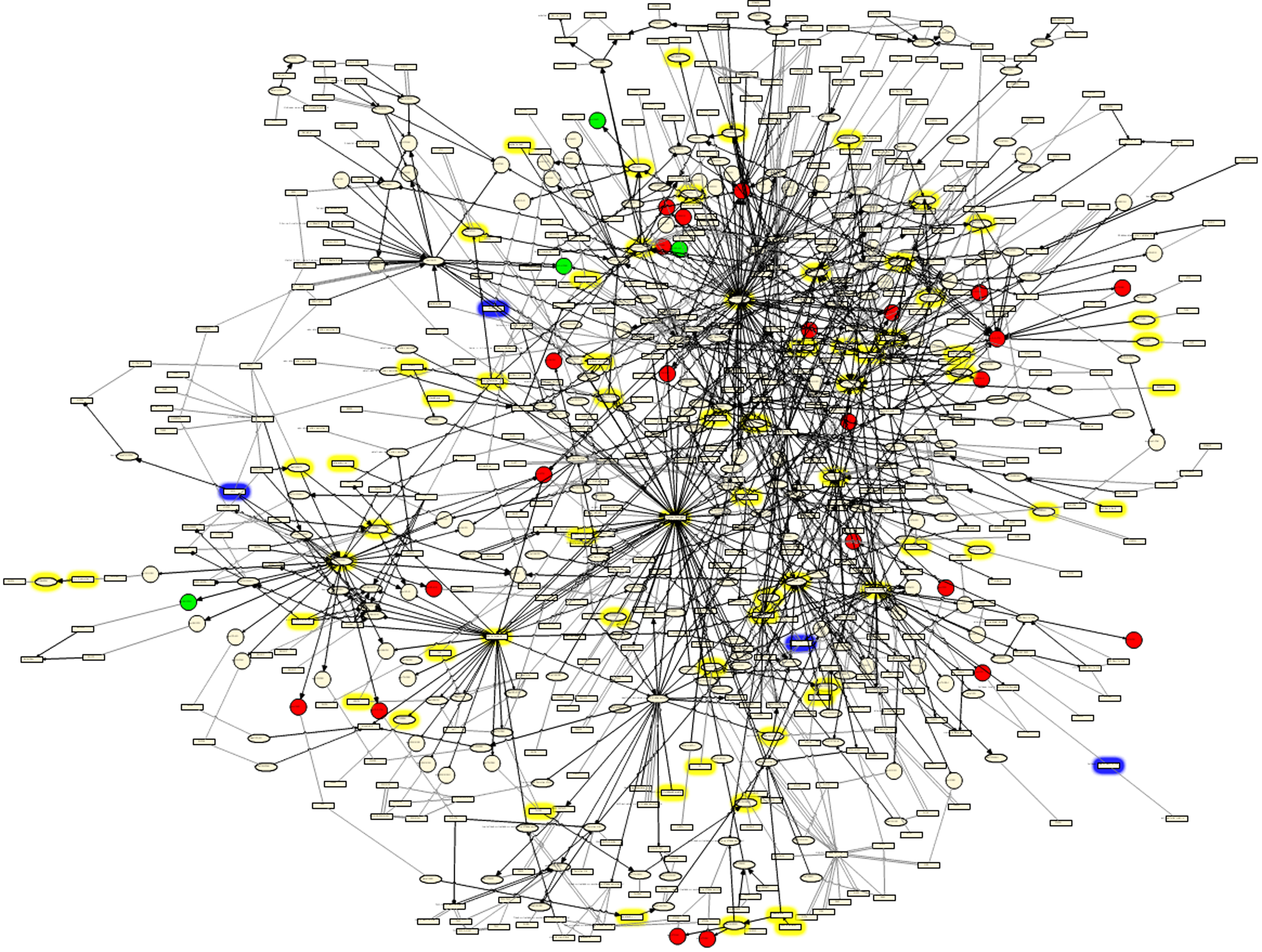

Supplement: Additional file 6 — Cellular Stress Network model colored for the hyperoxia data set. Red - node corresponds to observed increased mRNA; green - node corresponds to observed decreased mRNA; yellow halo - node is predicted by RCR to have increased activity; blue halo - node is predicted to have decreased activity. [file 1752-0509-5-168-S6.PNG]

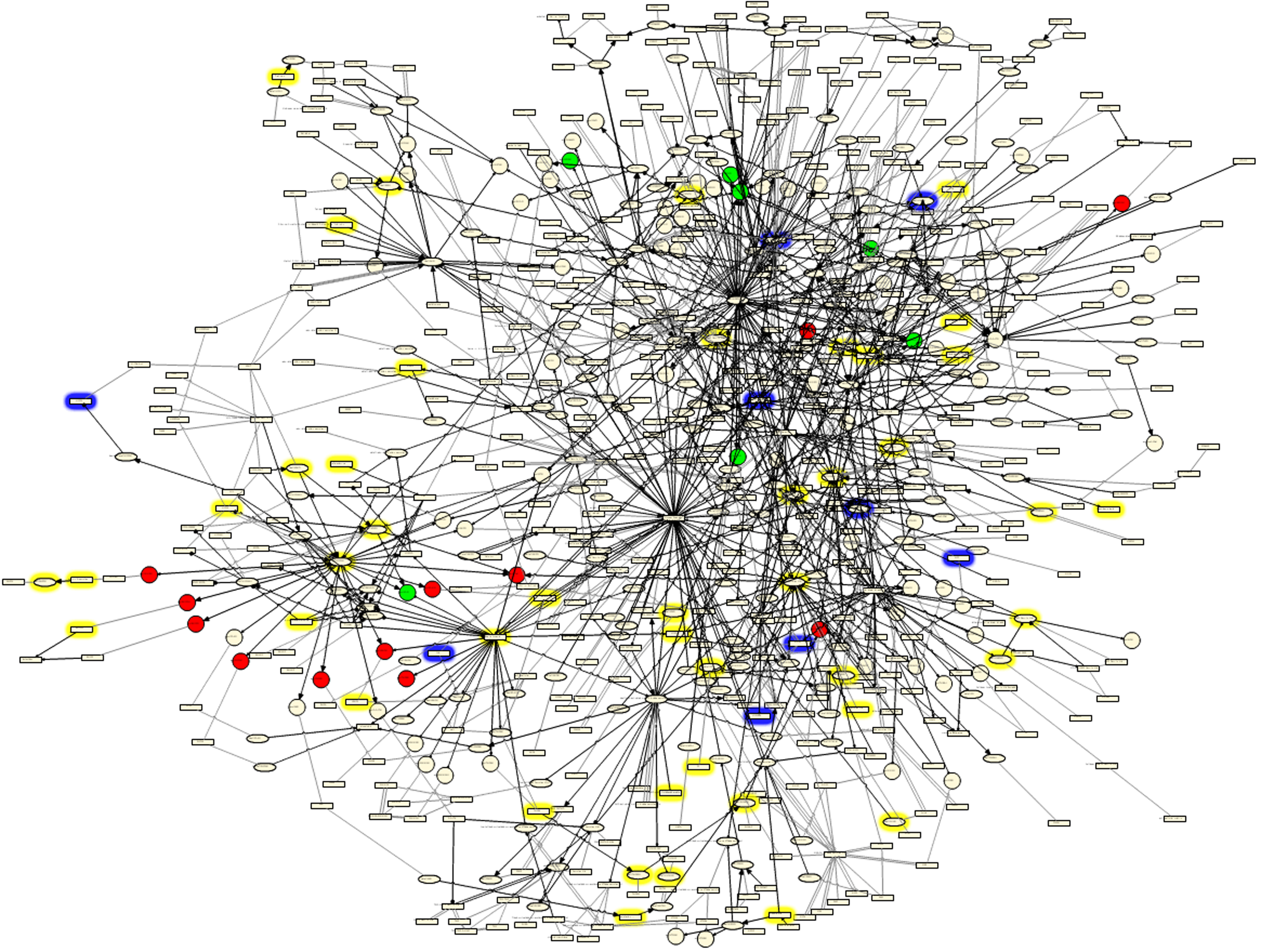

Supplement: Additional file 7 — Cellular Stress Network model colored for the hypoxia data set. Red - node corresponds to observed increased mRNA; green - node corresponds to observed decreased mRNA; yellow halo - node is predicted by RCR to have increased activity; blue halo - node is predicted to have decreased activity. [file 1752-0509-5-168-S7.PNG]

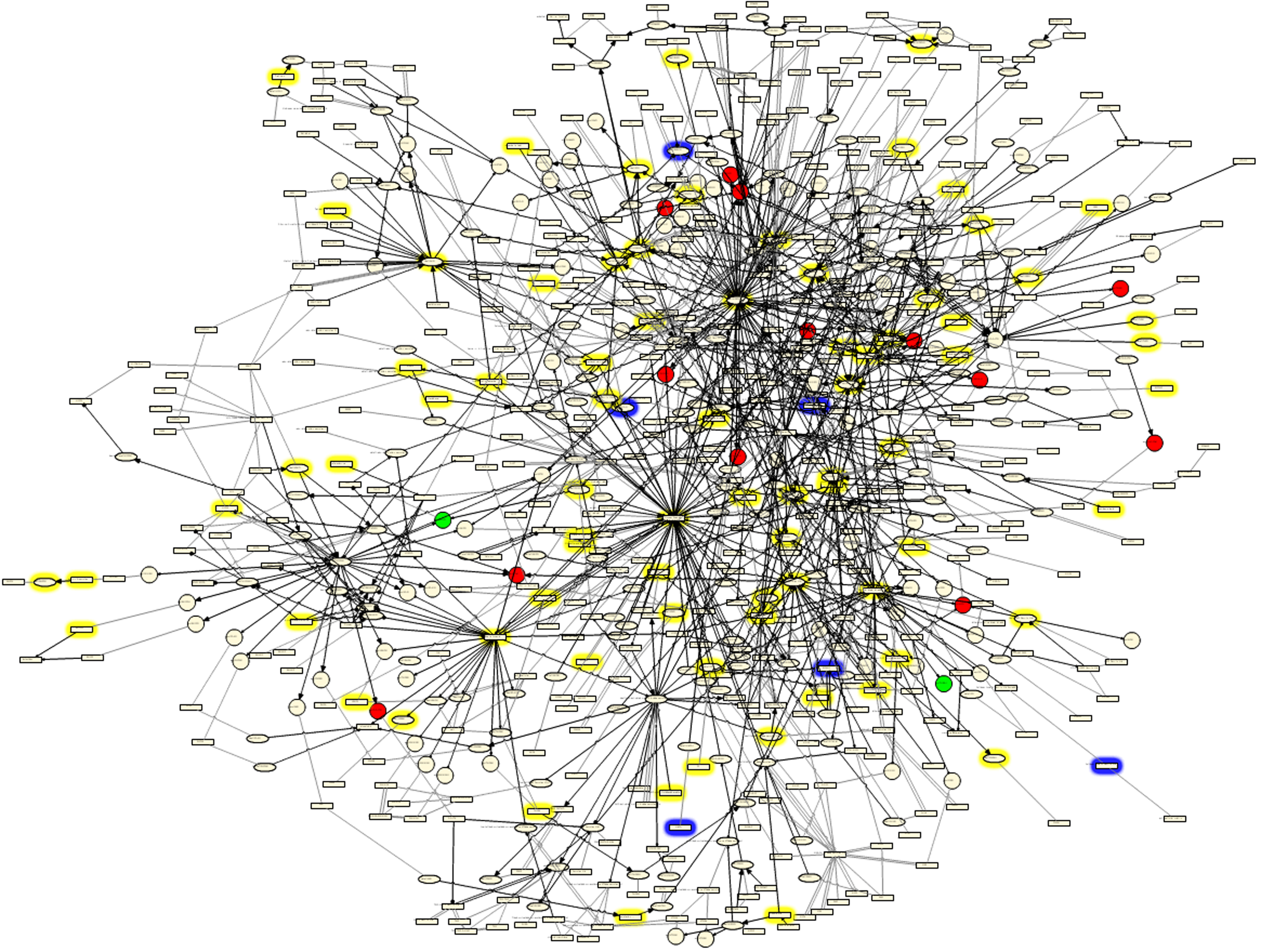

Supplement: Additional file 8 — Cellular Stress Network model colored for the OxPAPC data set. Red - node corresponds to observed increased mRNA; green - node corresponds to observed decreased mRNA; yellow halo - node is predicted by RCR to have increased activity; blue halo - node is predicted to have decreased activity. [file 1752-0509-5-168-S8.PNG]
